# Flt3 ligand expands *bona fide* innate lymphoid cell precursors *in vivo*

**DOI:** 10.1038/s41598-017-18283-0

**Published:** 2018-01-09

**Authors:** Sara M. Parigi, Paulo Czarnewski, Srustidhar Das, Christiane Steeg, Leonie Brockmann, Sara Fernandez-Gaitero, Victor Yman, Marianne Forkel, Charlotte Höög, Jenny Mjösberg, Lisa Westerberg, Anna Färnert, Samuel Huber, Thomas Jacobs, Eduardo J. Villablanca

**Affiliations:** 1 Immunology and Allergy Unit, Department of Medicine, Solna, Karolinska Institute and University Hospital, Stockholm, Sweden; 2 Department of Immunology, Bernhard-Nocht-Institut for Tropical Medicine, Hamburg, Germany; 3 Unit of Infectious Diseases, Department of Medicine, Solna, Karolinska Institute and University Hospital, Stockholm, Sweden; 4 Center for Infectious Medicine, Department of Medicine Huddinge, Karolinska Institutet, Stockholm, Sweden; 5 Unit for Inflammation, Gastroenterology and Rheumathology, Department of Medicine, Huddinge, Sweden; 6 Department of Clinical and Experimental Medicine, Linköping University, Linköping, Sweden; 7 Department of Microbiology, Tumor and Cell Biology, Karolinska Institutet, Stockholm, Sweden; 8 Department of Infectious Diseases, Karolinska University Hospital, Stockholm, Sweden; 9 Department of Medicine, University Medical Center Hamburg-Eppendorf, Hamburg, Germany

## Abstract

A common helper-like innate lymphoid precursor (CHILP) restricted to the innate lymphoid cells (ILC) lineage has been recently characterized. While specific requirements of transcription factors for CHILPs development has been partially described, their ability to sense cytokines and react to peripheral inflammation remains unaddressed. Here, we found that systemic increase in Flt3L levels correlated with the expansion of Lineage (Lin)^neg^α4β7^+^ precursors in the adult murine bone marrow. Expanded Lin^neg^α4β7^+^ precursors were *bona fide* CHILPs as seen by their ability to differentiate into all helper ILCs subsets but cNK *in vivo*. Interestingly, Flt3L-expanded CHILPs transferred into lymphopenic mice preferentially reconstituted the small intestine. While we did not observe changes in serum Flt3L during DSS-induced colitis in mice or plasma from inflammatory bowel disease (IBD) patients, elevated Flt3L levels were detected in acute malaria patients. Interestingly, while CHILP numbers were stable during the course of DSS-induced colitis, they expanded following increased serum Flt3L levels in malaria-infected mice, hence suggesting a role of the Flt3L-ILC axis in malaria. Collectively, our results indicate that Flt3L expands CHILPs in the bone marrow, which might be associated with specific inflammatory conditions.

## Introduction

FMS-like tyrosine kinase 3 ligand (Flt3L) is a hematopoietic cytokine that acts as a growth factor for early hematopoietic progenitors. By acting on its receptor Flt3 (CD135) and synergizing with other cytokines, Flt3L plays a pivotal role in promoting myeloid and lymphoid progenitors proliferation and differentiation^1,2^. Studies in mice have shown that lack of Flt3L results in deficient hematopoiesis, affecting primarily natural killer (NK) cells^3^, dendritic cells (DC)^3^, and common lymphoid progenitors (CLP)^1^. Besides regulating the development of immune cells during steady state conditions, Flt3L levels are raised upon inflammation, with the consequent expansion of hematopoietic cells. For instance, massive production of Flt3L by mast cells has been observed during malaria in mice^4^. Similarly, increase in serum Flt3L levels have been observed upon cytomegalovirus infection^5^ as well as in rheumatoid arthritis patients^6^. However, whether innate sensing of Flt3L plays a role in the pathogenesis of autoimmune diseases still remains unaddressed.

Innate lymphoid cells (ILCs) are a heterogeneous group of non-T, non-B lymphocytes comprising natural killer (NK) cells and distinct helper ILC subpopulations that differ from NK cells in terms of development and functions^7^. Based on their functional and transcriptional activity, helper ILCs are classified into three main subsets: Group 1 ILCs (ILC1s) express the transcription factor T-bet, produce interferon-γ (IFN-γ) and tumor necrosis factor alpha (TNF-α) and participate in intracellular bacterial immunity^7^; Group 2 ILCs (ILC2s) express the transcription factor GATA-3, secrete interleukin (IL) -4, IL-5, IL-9 and IL-13 and play a role in immunity to helminths and allergic inflammations^8^; and Group 3 ILCs (ILC3s) express the transcription factor RORγt, produce IL-17A and IL-22 and contribute to intestinal barrier homeostasis and protection towards extracellular bacteria^9^. ILC3s include lymphoid tissue inducer (LTi) cells, which are required for the development of secondary lymphoid structures^10^. This classification closely mirrors the T helper cell subsets; however, unlike their adaptive counterpart, ILCs lack antigen-specific receptor and do not require priming in secondary lymphoid organs^11^. Instead, ILCs quickly respond and get activated by environmental cues, such as cytokines, lipids, hormones and alarmins^12^.

ILCs have been identified in mouse and human, mainly in mucosal tissues, where they exert effector functions that can influence immune homeostasis or promote inflammation^13^. Although splenic ILCs promote antigen-specific T cell responses in mouse^14^, intestinal ILCs can restrict CD4 T cell responses against commensal bacteria^15,16^, hence suggesting context-dependent opposite roles. In addition, ILCs favor mucus production^8^, secretion of anti-microbial peptides^17^ and α(1,2)-fucosylation of intestinal epithelial cells^18^, therefore contributing to the homeostatic interaction between the host and the gut microbiota. ILCs have been also associated with pathology as seen by increased numbers of activated ILCs in tissue biopsies from patients suffering from inflammatory bowel disease (IBD)^19^. In line with this, using different murine models of chronic intestinal inflammation, ILC3s have been shown to accumulate in the inflamed colon and subsequently promote pathology^20–22^.

Lineage specification of ILCs mainly takes place during hematopoiesis. Although it belongs to the lymphoid lineage of hematopoiesis as T cells^23^, a separate developmental pathway has been proposed for ILCs in mouse^24^ and humans^25^. In the murine bone marrow (BM), downstream CLPs, a subset of early ILC progenitors (EILP) with NK and ILCs potential but lacking T and B cells potential is developmentally followed by a common helper ILCs precursor (CHILP)^7^. CHILPs are phenotypically defined in the murine BM by the lack of markers that identify other immune cell populations (CD5, B220, CD3, NK1.1, TER-119, CD19, Gr-1) and by the expression of IL-7 receptor (CD127) and the integrin alpha4 beta7 (α4β7). In addition, they differ from CLPs by the absence of CD135 and the expression of the transcriptional regulator inhibitor of DNA binding 2 (encoded by *Id2*)^7^. *In vitro* single cell clonal differentiation experiments from sorted CHILPs demonstrated their potential to generate one, two or all the three helper ILC subsets, suggesting a degree of heterogeneity and lineage pre-commitment among this population^7^. Downstream of CHILPs, up-regulation of the transcription factor PLZF generates a more restricted subset of ILC precursors (ILCP) committed to ILC1, ILC2 and ILC3 but lacking LTi potential. How ILC progenitors expand to give rise to all ILC subsets is still poorly understood. A recent report has shown that Flt3L promotes the expansion of NK, ILC2 and ILC3s by acting on lymphoid progenitors within the BM^26^. Whether inflammatory conditions raise Flt3L levels to generate ILCs *in vivo* is yet to be investigated.

Here, we show that increased levels of systemic Flt3L are associated with expansion of *bona fide* CHILPs in the BM. By using adoptive transfer experiments, we demonstrated that Flt3L-mediated expansion does not alter the ability of CHILPs to selectively give rise to ILCs *in vivo*. In order to investigate if, under disease conditions, rising levels of Flt3L induce demanded “ILC-poiesis”, we analyzed Flt3L systemic levels and CHILP expansion in the BM upon inflammation. Although we did not observe any correlation during intestinal inflammation, we showed that CHILPs are expanded upon elevated Flt3L serum levels in malaria-infected mice. Collectively, our results suggest that CHILPs expand in response to Flt3L and this axis may play a role in some inflammatory conditions.

## Results

### Flt3L-producing tumors expand α4β7^+^ ILC precursors in the BM

**Figure 1:**
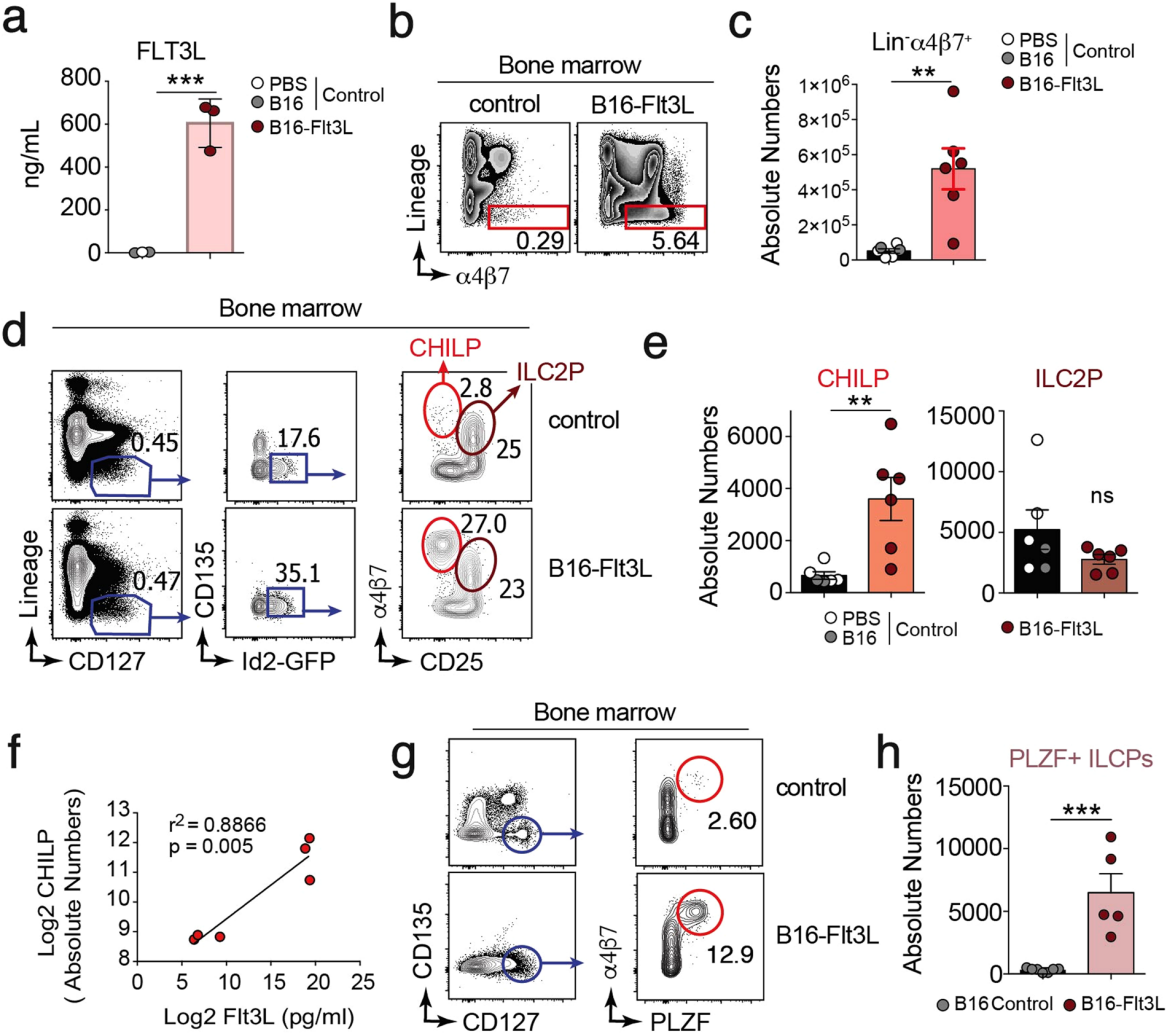
Flt3L-producing tumors expand CHILPs in the BM. Mice were injected with 2 × 10^6^ B16-Flt3L or B16/PBS as a control. Two weeks after tumor injection, Flt3L serum levels were analyzed by ELISA and CHILPs and ILC2P were analyzed by FACS on BM single-cell suspension. (**a**) Flt3L serum levels in B16/PBS and B16-Flt3L injected mice (*n* = 3/group, 2 experiments). Representative dot plots (**b**) and total number (**c**) of Lin^−^α4β7^+^ cells in the BM of wild-type mice injected with B16-Flt3L or B16/PBS (*n* = 6/group, 3 experiments). Representative dot plots (**d**) and absolute numbers (**e**) of CHILPs and ILC2P in the BM of *Id2* ^*Gfp*/+^ mice injected with B16-Flt3L or B16/PBS (*n* = 6/group, 3 experiments). (**f**) Correlation plot between Flt3L serum levels determined by ELISA and BM CHILPs absolute numbers determined by FACS in mice treated with B16-Flt3L or B16 (*n* = 6, 2 experiments). Representative dot plots (**g**) and absolute numbers (**h**) of ILCP in the bone marrow of wild type mice injected with B16-Flt3L or B16 cells (*n* = 5 to 7/group, 3 experiments). *P < 0.05; **P < 0.01; ns, not significant; Student’s *t*-test (**c**,**e**,**h**), Linear regression with Pearson’s correlation analysis (**f**). Error bars represent SEM in all panels.

To examine the effect of Flt3L levels on hematopoiesis, wild type (WT) C57BL/6 mice were subcutaneously injected with B16 melanoma cell line that constitutively secretes Flt3L (B16-Flt3L) and analyzed after 14 days. As expected, systemic serum Flt3L levels **(**Fig. 1a **)** and splenic differentiated (CD11c^+^ MHC-II^+^) and immature (CD11c^+^ MHC-II^−^) dendritic cells (DCs) numbers **(**Supplementary Fig. 1a and b) were increased in B16-Flt3L injected mice compared to PBS or B16 injected controls. In the bone marrow, we found 10-fold expansion of Lineage (Lin; CD11c, TER-119, CD3, CD19, CD11b, NK1.1)^neg^ BM cells expressing the integrin α4β7 in B16-Flt3L-injected compared to control mice (Fig. 1b and c). To address if α4β7^+^ expanded cells were phenotypically similar to CHILPs, defined as Lin^neg^ CD127^+^ CD135^neg^ Id2^+^ α4β7^+^ CD25^neg^ cells^7^, we injected B16-Flt3L in Id2 reporter mice (*Id2* ^*Gfp*/+^). Analysis of BM cells suspension showed a dramatic increase in frequencies and absolute numbers of Lin^neg^ CD127^+^ CD135^neg^ Id2^+^ α4β7^+^ CD25^neg^ CHILPs, when compared to mice injected with either B16 cells or PBS as a control (Fig. 1d and e). Moreover, CHILPs expansion within the BM positively correlated with serum Flt3L levels (Fig. 1f), suggesting dose-dependency. By contrast, ILC2-committed precursors (ILC2P) defined as Lin^neg^ CD127^+^ CD135^neg^ Id2^+^ α4β7^+^ CD25^+^, remained unchanged (Fig. 1d and e). Of note, B16-Flt3L injection resulted in non-detectable CD135^+^ (Flt3 receptor) expressing cells (Fig. 1d **)**, hence suggesting downregulation of the receptor upon activation. Common ILC precursors (ILCPs), characterized as Lin^neg^ CD135^−^ CD127^+^ α4β7^+^ PLZF^+^, give rise to all ILC subsets but conventional NK cells (cNK) and lymphoid tissue inducer (LTi) cells, thus being considered a more restricted ILC precursor downstream of CHILPs^27,28^. Concomitant with the expansion of CHILPs, PLZF^+^ ILCPs numbers were increased in the bone marrow of B16-Flt3L injected mice compared to its control counterpart (Fig. 1g and h). In order to rule out any concerted effect of the tumor and Flt3L overexpression, WT mice were injected intraperitoneally with 10 μg of recombinant Flt3L for 10 consecutive days. Similar to what was observed with B16-Flt3L, Flt3L cytokine administration resulted in significant increase in CHILPs and ILCPs, but not of ILC2P numbers in the bone marrow over control mice **(**Supplementary Fig. 1c and d).

**Figure 2:**
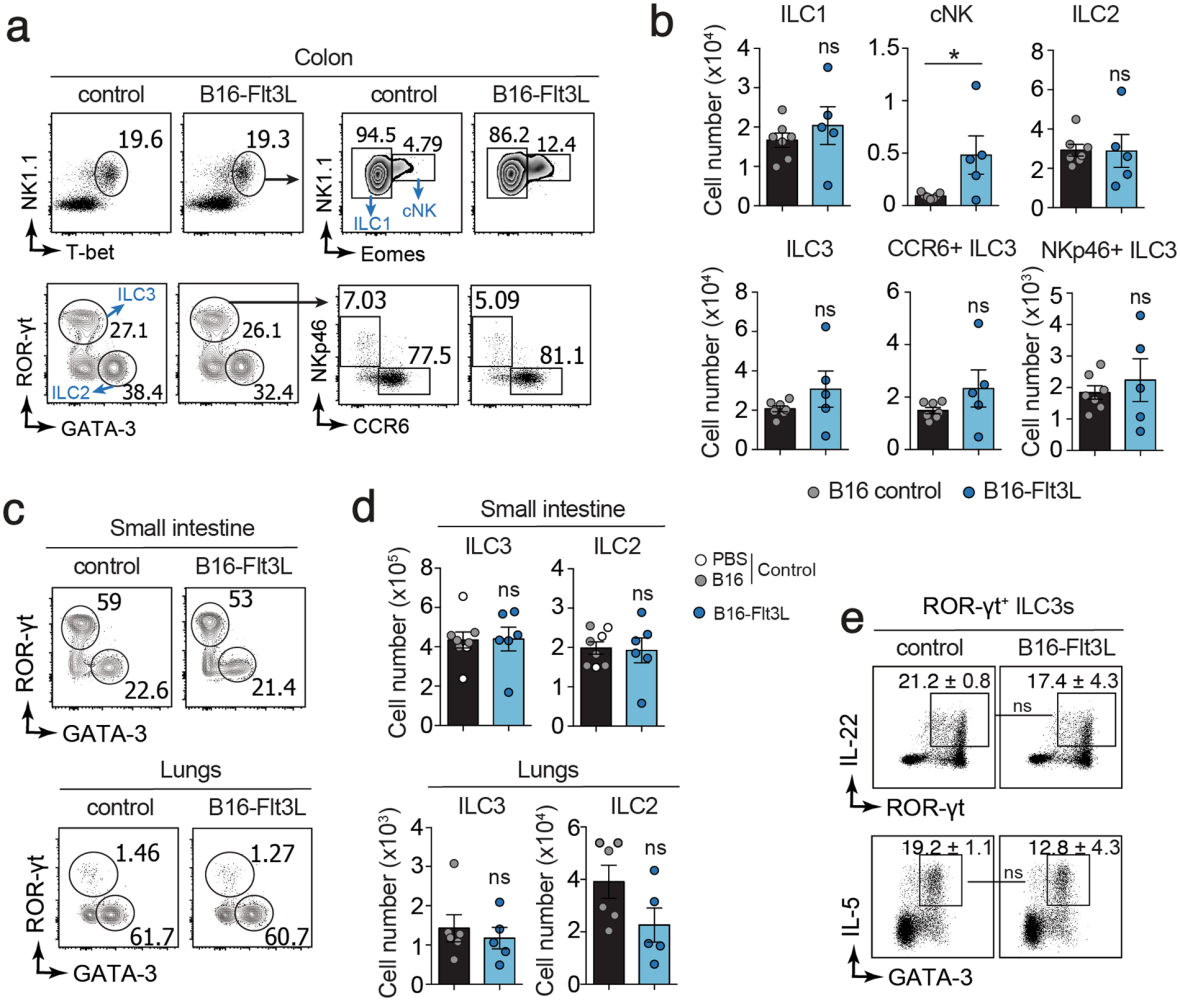
Flt3L-producing tumors do not expand mature ILCs in periphery. Mice were injected with 2 × 10^6^ B16-Flt3L or B16/PBS as a control. Two weeks after tumor injection, ILCs were analyzed by FACS in the lungs, small intestine and colonic lamina propria. (**a**) Representative dot plots showing NK1.1^+^T-bet^+^Eomes^−^ ILC1, NK1.1^+^T-bet^+^Eomes^+^ cNK, GATA-3^+^ ILC2, RORγt^+^ ILC3 and NKp46^+^ and CCR6^+^ ILC3 subsets gated on CD45^+^ Lin (CD3, CD19)^neg^ CD90^+^ cells in the colonic lamina propria and (**b**) quantification of absolute numbers (*n* = 5 to 7/group, 3 experiments). Representative dot plots (**c**) and absolute numbers (**d**) of ILC2 and ILC3 in the small intestine lamina propria and lungs of B16-Flt3L and B16/PBS injected mice (*n* = 5 to 8/group, 3 experiments). (**e**) Representative dot plots of IL22^+^ RORγt^+^ ILC3 and IL-5^+^ GATA-3^+^ ILC2 in the small intestine of B16-Flt3L and B16 injected mice (*n* = 3 to 4, 2 experiments). *P < 0.05; ns, not significant; Student’s *t*-test (**b**,**d**,**e**). Error bars represent SEM in all panels.

To investigate whether CHILP expansion observed in B16-Flt3L-injected mice results in increased number of ILCs, we analyzed mature ILCs subsets in different peripheral tissues. We did not detect differences in the frequencies or numbers of ILC1s (defined as Lin (CD3, CD19)^neg^ CD45^+^ CD90^+^ NK1.1^+^ T-bet^+^ Eomes^−^), ILC2s (defined as Lin^neg^ CD45^+^ CD90^+^ GATA-3^+^), ILC3s (defined as defined as Lin^neg^ CD45^+^ CD90^+^ RORγt^+^) and ILC3s subsets (NKp46^+^ or CCR6^+^) **(**Supplementary Fig. 2a) in the colonic **(**Fig. 2a and b) and small intestine lamina propria **(**Fig. 2c and d, Supplementary Fig. 2b), lungs **(**Fig. 2c and d) and liver (Supplementary Fig. 2b) between B16-Flt3L injected and B16-injected control mice. Conversely, in line with previous reports^3^, conventional NK (cNK) cells were expanded in the colon of B16-Flt3L bearing mice **(**Fig. 2a and b). In addition, we compared the functional properties of mature ILCs from B16-Flt3L-injected and control mice in the small intestine by analyzing cytokine production. We did not observe any difference in the ability to produce IL-22 by ILC3s and IL-5 by ILC2s **(**Fig. 2e **)** irrespective of the experimental groups. Altogether our data associate increased Flt3L levels with expansion of phenotypically defined CHILPs in the BM without affecting peripheral ILCs numbers or function.

### Flt3L-expanded Id2^+^α4β7^+^ BM cells are *bona fide* CHILPs

**Figure 3:**
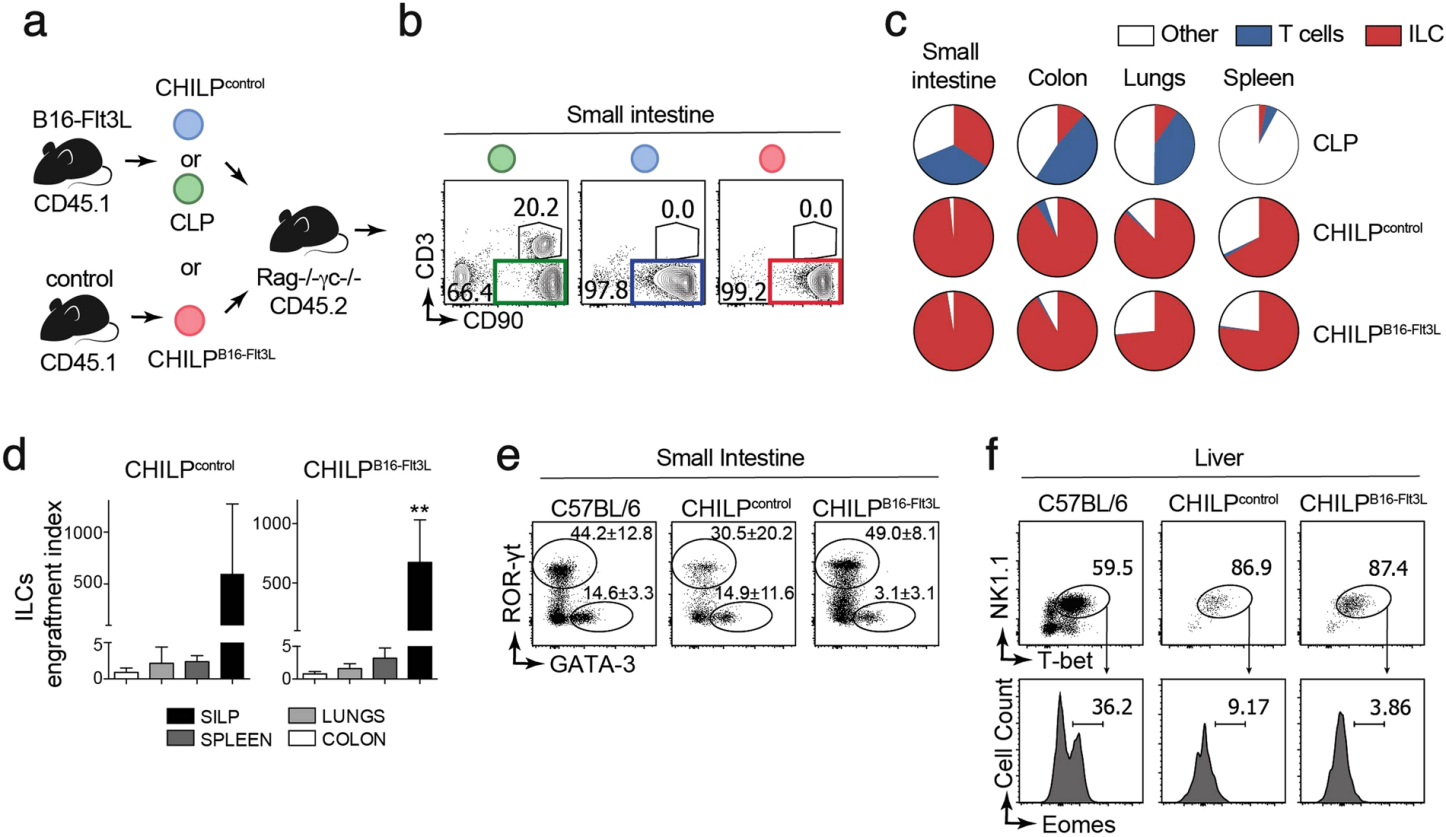
Flt3L expands *bona fide* CHILPs. FACS-sorted CHILPs were isolated from PBS- (CHILP^control^) or B16-Flt3L-injected (CHILP^B16-Flt3L^) *Id2* ^*Gfp*/+^ mice. CLPs were isolated from PBS-injected mice. The indicated highly purified cell populations were adoptively transferred into congenic alymphoid recipients, which were analyzed 5 to 10 weeks later. (**a**) Schematics of the experimental design. (**b**) Representative dot plots showing the analysis of ILCs (CD45.1^+^ CD90^+^ CD3^−^) and T cells (CD45.1^+^ CD90^+^ CD3^+^) from the small intestine lamina propria (SILP). Displayed plots are gated on CD45.1^+^ donor-derived cells. (**c**) Pie plots showing the frequencies of ILCs, T cells and the rest out of all the donor-derived cells (CD45.1^+^) from different tissues (*n* = 4 for CHILP^B16-Flt3L^ group; *n* = 2 for CHILP^Control^ group; *n* = 3 for CLP group, 2 experiments). (**d**) Engraftment index of CHILP^Control^ and CHILP^B16-Flt3L^ in different organs (*n* = 2 to 4, 2 experiments). (**e**) Representative dot plots of RORγt^+^ ILC3 and GATA-3^+^ ILC2 gated on total intestinal donor-derived ILCs (*n* = 4–6/group, 3 experiments). Representative dot plot of RORγt^+^ ILC3 and GATA-3^+^ ILC2 gated on total intestinal ILCs from a non-transferred wild-type C57BL/6 mouse is shown as a control. (**f**) Representative dot plots showing NK1.1^+^T-bet^+^ cells gated on donor derived CD90^+^ cells in the liver (non-transferred C57BL/6 wild type is shown as a control) and representative histograms showing Eomes^+^ cNK. ns, not significant; **P < 0.01; one-way ANOVA with Bonferroni’s multiple comparisons test (**d**). Average and standard deviation are displayed in all panels.

Since peripheral ILC numbers were unchanged (despite the ~5-fold expansion of Lin^neg^ CD127^+^ CD135^neg^ Id2^+^ α4β7^+^ CD25^neg^ precursors) we tested the possibility that expanded CHILPs were non-viable, non-functional or they were contaminated with a large proportion of CLPs that internalized CD135 upon sensing of Flt3L. To address this question, we adoptively transferred highly purified CD45.1^+^Lin^neg^ CD127^+^ CD135^neg^ Id2^+^ α4β7^+^ CD25^neg^ cells from either control (CHILP^control^) or B16-Flt3L-injected mice (CHILP^B16-Flt3L^) into congenic CD45.2^+^ alymphoid (*Rag2* ^−/−^ *Il2rg* ^−/−^) recipients (Supplementary Fig. 3a and Fig. 3a). In addition, purified CLPs (Lin^neg^ CD127^+^ CD135 ^+^ Id2^neg^) were adoptively transferred into congenic *Rag2* ^−/−^ *Il2rg* ^−/−^ recipients as positive control for T cell and ILC differentiation (Supplementary Fig. 3a and Fig. 3a). Five to ten weeks after transfer, the presence of donor-derived cells (CD45.1^+^) was evaluated in the small intestine lamina propria of recipient mice. Both CHILP^control^ and CHILP^B16-Flt3L^ gave rise to more than 98% CD90^+^Lineage (CD11c, Gr-1, CD64, TER-119, CD19)^neg^ cells without detectable Lin^+^CD90^neg^ cells, indicating that CHILPs differentiated neither into non-lymphoid nor B cells (Supplementary Fig. 3b). We then analyzed whether CD90^+^Lin^neg^ cells were T cells and/or ILCs. As expected, injection of CLPs gave rise to T cells (CD90^+^CD3^+^) and ILCs (CD90^+^CD3^neg^) (Fig. 3b). On the other hand, CHILP^control^ and CHILP^B16-Flt3L^ gave rise uniquely to ILCs, (Fig. 3b), demonstrating that Flt3L-expanded Lin^neg^ CD127^+^ CD135^neg^ Id2^+^ α4β7^+^ CD25^neg^ cells were *bona fide* CHILPs. Next, we further characterized Flt3L-expanded CHILPs in other organs beyond the small intestine. Analysis across different tissues showed that both CHILP^control^ and CHILP^B16-Flt3L^ gave rise mostly to ILCs (Fig. 3c). Thus, regardless of the tissue, Flt3L-expanded CHILPs, were comparable to untreated CHILPs in their capacity to differentiate to ILCs *in vivo*.

Previous studies have shown that the progeny of untreated CHILPs adoptively transferred into alymphoid recipients was mainly found in the small intestine, with very few donor-derived cells present in secondary lymphoid organs^7^. Similarly, when we analyzed the number of mature ILCs per injected CHILP (engraftment index), we observed higher engraftment potential in the small intestine compared to the other tissues (Fig. 3d). Whether the enhanced ability of CHILPs to reconstitute mostly the small bowel depends on the expression of the gut homing molecule α4β7 requires further investigation.

Next, we investigated the potential of Flt3L–expanded CHILP to differentiate into different ILCs subsets. Similar to CHILP^control^, CHILP^B16-Flt3L^ gave rise to RORγt^+^ ILC3s and GATA3^+^ ILC2s in the small intestine (Fig. 3e). In line with previous results^7^, donor-derived T-bet^+^ ILC1s were almost undetectable in the small intestine (Supplementary Fig. 3c) but they were highly prevalent in the liver of recipient mice. While both T-bet^+^Eomes^−^ ILC1s and T-bet^+^Eomes^−^ cNK can be detected in the liver of non-transferred wild type mice (C57BL/6), both CHILP^control^ and CHILP^B16-Flt3L^ gave rise exclusively to T-bet^+^Eomes^−^ ILC1s (Fig. 3f), indicating that Flt3L-expanded CHILPs have the potential to differentiate to all ILC lineages but cNK *in vivo*. Altogether, we concluded that Flt3L promoted the expansion of *bona fide* CHILPs in the BM, without altering their helper ILCs differentiation potential *in vivo*.

### Unchanged serum Flt3L levels and CHILP numbers during intestinal inflammation

**Figure 4:**
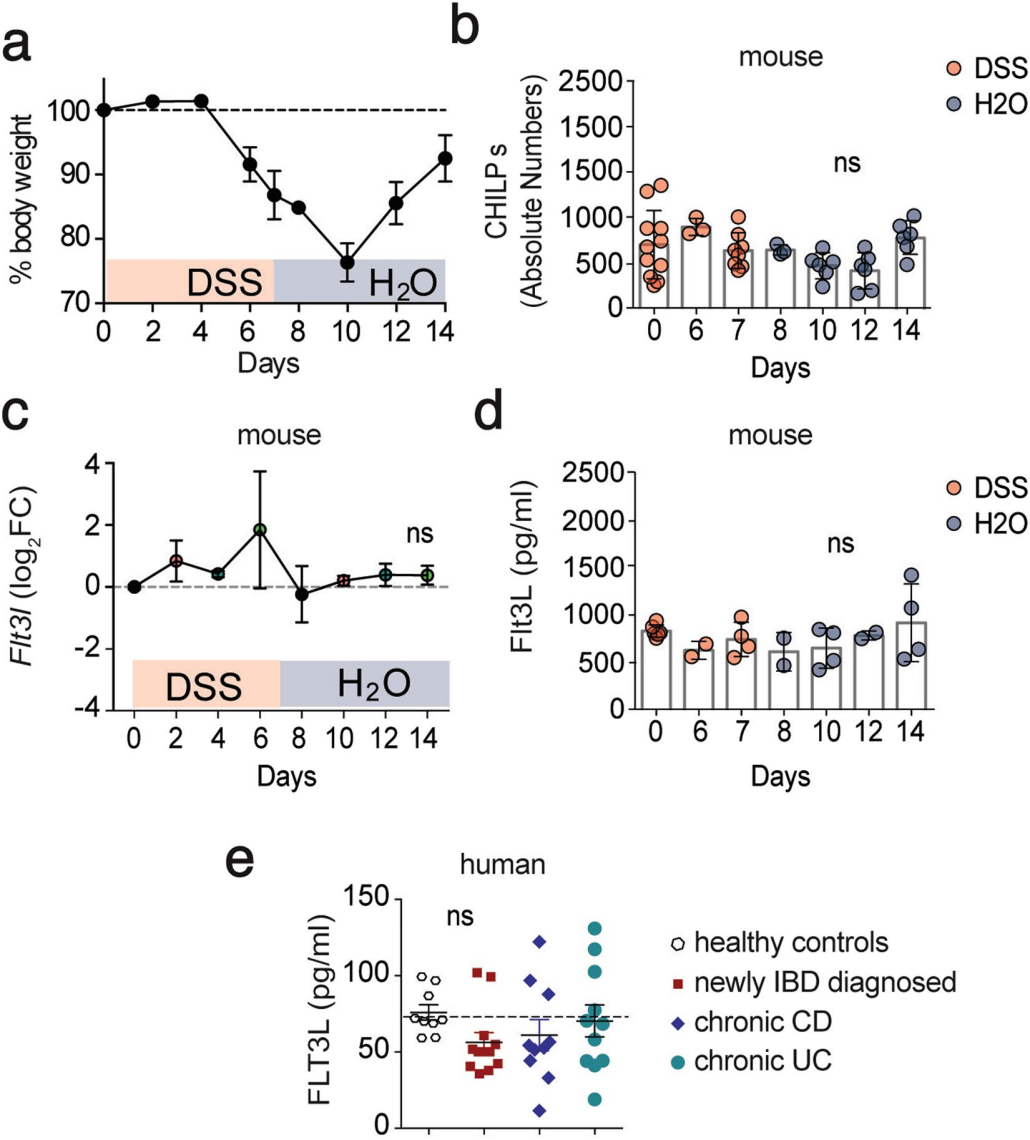
Unchanged serum Flt3L levels and CHILP numbers during intestinal inflammation. (**a**–**d**) Wild type mice were treated for 7 days with 2.5% DSS in drinking water followed by 7 days of regular water. Mice were sacrificed and analyzed at the indicated time points. (**a**) Body weight monitored during the course of the treatment. Body weight of mice sacrificed at the different time points are indicated (*n* = 3/time point). (**b**) Quantification of CHILPs numbers in the BM of mice treated with DSS at the indicated time point (*n* = 3 to 11/time point, 3 experiments) (**c**) mRNA transcript expression for *Flt3l* in colon was measured by quantitative PCR at the indicated time point (*n* = 3/time point) (**d**) Serum levels of Flt3L determined by ELISA at the indicated time points (*n* = 2 to 6/time point, 2 experiments). (**e**) FLT3L plasma levels in healthy controls, newly diagnosed IBD patients and chronic Crohn’s disease (CD) and Ulcerative colitis (UC) patients. Each dot represents an individual patient. *P < 0.05; **P < 0.01; ns, not significant; two-way ANOVA with Bonferroni’s multiple comparisons test (**b**,**d**) and one-way ANOVA with Bonferroni’s multiple comparisons test (**e**). Error bars represent SD in all panels.

Our findings that increased systemic levels of Flt3L expand ILCs precursors in the BM, raised the question whether this mechanism plays a role during inflammatory conditions. Since dysregulation of the ILC pool has been observed in inflamed colonic tissue of IBD patients^19,29,30^ and in mouse models of chronic intestinal inflammation^20,31^, we explored the possibility that Flt3L-mediated ILC-poiesis might account for these observations. First, we used a model of chemically induced colitis in which WT mice were exposed to 7 days of 2.5% dextran sulfate sodium (DSS) in drinking water followed by 7 days of regular water. As expected, we observed body weight loss starting by day 6 after DSS treatment and reaching its maximum 3 days after the DSS was removed and replaced by water (Fig. 4a). By day 10 of the experiment, mice started to recover their body weight reaching almost complete recovery by day 14 (Fig. 4a). Analysis of the BM showed non-significant differences in CHILPs numbers through the course of the experiment (Fig. 4b). We next sought to investigate whether intestinal inflammation induces changes in Flt3L levels. We first analyzed the levels of *Flt3l* transcripts in total colonic tissues during the course of the experiment. Our longitudinal transcriptomic analysis did not show any significant difference in *Flt3l* transcripts in the proximal colonic tissues during the course of the experiment (Fig. 4c). Flt3L is present in two biologically active different forms, membrane-bound or cleaved and secreted^32^. Since we did not detect any transcriptional alteration, we tested if intestinal inflammation was promoting the cleavage and systemic release of Flt3L. However, similar to colonic transcript levels, we did not detect any significant increase or decrease of Flt3L protein levels in the serum of mice treated with DSS **(**Fig. 4d). In order to validate that soluble Flt3L remains unchanged during chronic intestinal inflammation and to avoid being biased by the mouse model we exploited, we measured FLT3L in plasma from newly diagnosed or established IBD patients. In agreement with our mouse data, FLT3L plasma levels were not altered in newly diagnosed IBD patients nor in chronic Crohn’s disease (CD) and ulcerative colitis (UC) patients compared to healthy donors (Fig. 4e). Taken together, these results, combining human and mouse data, show that soluble Flt3L systemic concentration is not altered during intestinal inflammation and at least in mice, CHILPs size in the BM seems to be stable through the course of colitis.

### CHILPs expand in the BM during malaria infection

**Figure 5:**
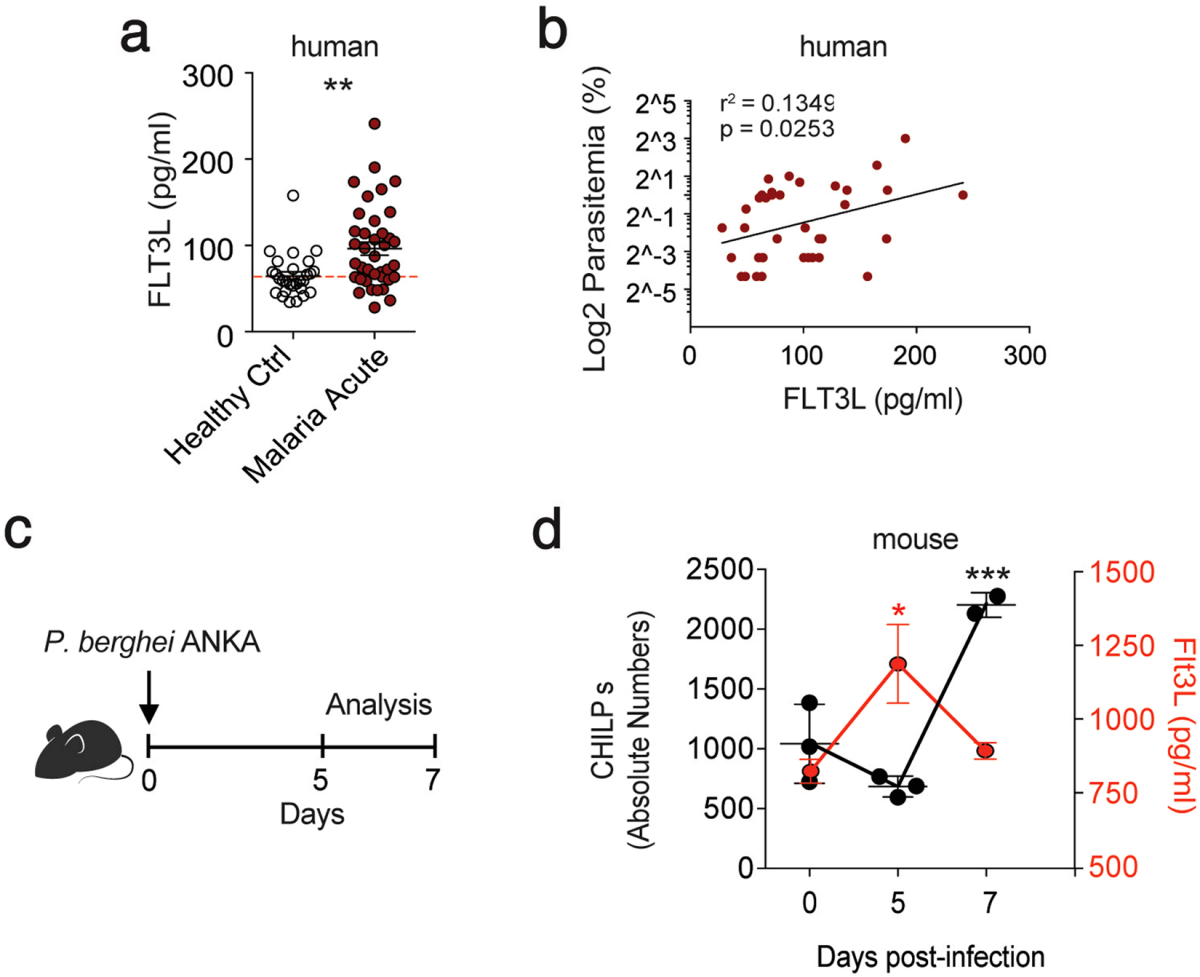
CHILPs expand in the BM during malaria. (**a**) Plasma FLT3L in healthy controls and patients suffering from acute *P. falciparum* malaria. Each dot represents an individual healthy control (*n* = 28) or *Plasmodium falciparum* infected patient (acute malaria; *n* = 39). Dashed line represents the average Flt3L levels in healthy donors. (**b**) Correlation plot between plasma FLT3L levels and percentage of parasitemia in patients suffering from acute malaria. Each dot represents an individual patient. Coefficient of determination was calculated by Pearson’s correlation analysis. (**c**,**d**) Wild type mice were infected with *P. berghei* ANKA and were analyzed at day 5, and 7 after infection. (**c**) Schematics of the experimental design. (**d**) Quantification of CHILPs number in the BM (black dots) and Flt3L serum levels (red dots) at the indicated time points (*n* = 3 at day 0 and 5; *n* = 2 at day 7). *P < 0.05; **P < 0.01; ***P < 0.001; ns, not significant; one-way ANOVA with Bonferroni’s multiple comparisons test (**d**). Error bars represent SD for CHILPs numbers and SEM for Flt3L serum levels.

It has been reported that, during acute malaria infection, CLPs undergo a transient depletion, whereas a population of IL-7R^+^c-Kit^hi^ myelolymphoid progenitors gets expanded in the bone marrow^33^. A more recent study has shown increased plasma FLT3L levels in acute malaria patients^4^. Since CHILPs express IL-7R (CD127) and c-Kit^7^, we hypothesized that CHILPs might contribute to the expanded pool of CD127^+^ progenitors in the BM and that rising levels of systemic FLT3L precedes this process. We first confirmed in our cohort of malaria patients that plasma levels of FLT3L are significantly increased in a group of *Plasmodium falciparum* infected adults compared to healthy controls (Fig. 5a). In addition, we observed a significant positive correlation between plasma FLT3L concentration and level of parasitemia at diagnosis (Fig. 5b). Next, we aimed to investigate if increased FLT3L levels upon malaria infection are associated with expansion of CHILPs in the BM. Since we do not have access to BM from acute malaria patients, we infected C57BL/6 mice with *Plasmodium berghei* ANKA and we analyzed the frequencies of CHILPs and serum Flt3L levels at day 0, 5, and 7 post-infection (Fig. 5c). We found that serum Flt3L levels transiently increase three-fold by day 5 post-infection when compared to uninfected animals. Similarly, we observed a transient 2-fold expansion of CHILPs in the BM at day 7 post-infection (Fig. 5d), which was preceded by the peak of Flt3L serum levels. The sequential increase of Flt3L serum levels followed by CHILPs expansion in the BM suggests that the Flt3L-CHILPs axis might play a role in the context of malaria infection.

## Discussion

Flt3L is a crucial regulator of lymphoid^1^ and myeloid^34,35^ progenitors pool in the BM under steady state conditions. Inflammation- or experimentally-induced perturbation of systemic Flt3L levels results in emergency demanded hematopoiesis in the BM, characterized by the expansion of early Flt3^+^ progenitors and expansion of dendritic cells in periphery^36^. However, whether inflammation-driven increase in Flt3L regulates ILCs lymphopoiesis remains unknown. In this study, we showed that a transient systemic increase of Flt3L causes the expansion of *bona fide* ILCs progenitors in the BM without affecting their expansion and differentiation potential *in vivo*. We also demonstrated that intestinal inflammation, where ILCs are known to play a role, is not characterized by the increase of Flt3L nor by the expansion of ILCs precursors in the BM. Conversely, we showed that, upon malaria infection, rising levels of Flt3L precede the expansion of CHILPs in the BM.

ILCs lymphopoiesis relies on a recently identified committed progenitor population, termed CHILPs, that resides in the BM^7^. Several transcription factors have been proven necessary for CHILP development, including *Tox, Id2, Nfil3* ^28^. However, little is known about the ability of soluble cytokines to regulate CHILP hematopoiesis. The current model for ILCs precursors ontogenesis, during fetal development, proposes a step-wise up-regulation of α4β7 followed by the down-regulation of Flt3 (Flt3L receptor) from the CLP stage that ultimately converges towards CHILPs generation^37^. Whether Flt3 down-regulation on CHILPs is the result of Flt3L binding, signaling and subsequent internalization of the receptor still remains to be addressed. Suggesting a role for Flt3L in skewing BM lymphopoiesis towards the ILCs lineage, *in vitro* studies showed that sorted Rag2^−/−^ CLP, cultured in the presence of Flt3L, were more prone to generate ILC3s^26^. During the preparation of this manuscript, a study by Finke’s group described reduced numbers of CHILPs in the adult Flt3L^−/−^ BM compared to their WT counterpart^26^. Our study further corroborated these findings by showing that, in response to a transient systemic increase of Flt3L, CHILPs undergo expansion in the BM. In the murine BM, CHILPs can differentiate into more committed precursors, as ILC2P that selectively give rise to ILC2s^38^. Of note, differently from what was reported in Flt3L^−/−^ mice, we did not observe any alteration of ILC2P numbers in the BM of mice injected with B16-Flt3L. This discrepancy might be explained by the fact that genetic ablation is more penetrant compared to our short-term exposure to Flt3L.

Interestingly, despite the increased numbers of ILCs precursors in the BM of B16-Flt3L treated mice, we did not observe expansion of mature ILCs in periphery. Previous studies have shown reduced peripheral ILCs numbers in the setting of congenital Flt3L deficiency, as opposed to mice with constitutive overexpression of Flt3L where ILCs expansion was reported^26^. However, differently from genetically engineered mice, our short-term Flt3L exposure might not be sufficient to affect the pool of mature ILCs. This phenomenon is in line with previous findings supporting the low turnover of tissue-resident ILCs^39^. An alternative explanation is an intrinsic impairment in CHILPs differentiation potential upon Flt3L-mediated expansion. However, this seems to be unlikely since we observed efficient differentiation potential *in vivo*. This is particularly relevant as it provides a model to expand a rare BM population to be used as a tool to study the biology of ILCs *in vivo*.

We further elucidated the role of the Flt3L-CHILPs axis in disease conditions. ILCs, as immune gatekeepers at mucosal sites, may be important players in the pathogenesis of intestinal inflammation^13,22^ and infection^20,40,41^. In agreement, expansion of pathogenic IL17-producer ILCs has been reported in the inflamed intestine of Crohn’s disease patients^19^. However, we did not observe significant changes in BM-resident CHILPs numbers during DSS-induced intestinal inflammation and recovery. Likewise, we did not observe changes in Flt3L serum levels during the same experiment. Consistently, we did not detect any alteration in FLT3L systemic levels in patients with newly diagnosed or chronic IBD, thus suggesting that release of Flt3L is not a hallmark of intestinal inflammation.

Differently from intestinal inflammation, malaria infection is characterized by increased levels of systemic Flt3L that is produced by mast cells in response to sensing of uric acid crystals accumulation^4^. Our data further corroborated a positive correlation between systemic Flt3L levels and the intensity of infection, in both mice and human. Interestingly, in line with our initial results showing an Flt3L-mediated expansion of CHILPs, we detected expansion of CHILPs in the BM of *Plasmodium berghei*-infected mice, following the peak of Flt3L accumulation. Altogether, our data suggest a role for the Flt3L-CHILPs axis upon malaria infection. However, the effect of CHILPs accumulation or the functional consequences of blocking this axis upon malaria infection still remain to be addressed. A previous study has linked *Plasmodium* infection in mice with profound alteration in BM hematopoiesis^33^. In particular, infection with *P. chabaudi* caused a defective T and B cells lymphopoiesis coupled by the emergence of a new class of infection-induced c-Kit^+^ CD127^+^ myelolymphoid precursors^33^. The progeny of these newly identified progenitors was shown to be crucial for the clearance of infected erythrocytes^33^. Of note, CHILPs in the murine BM are characterized by the expression of CD127 and c-Kit, but whether CHILPs contribute to the expanding pool of this protective infection-induced class of progenitors is a subject for future investigation.

In summary, our study demonstrates that Flt3L acts as an important regulator of ILCs lymphopoiesis and it can be used as a tool to expand and study ILCs precursors in the BM. In addition, we showed how ILCs precursors sense and react to peripheral inflammation, characterized by increased systemic levels of Flt3L. Particularly, in malaria, we observed increased systemic Flt3L levels preceding CHILPs expansion in the BM. Altogether, our results open up a new area of investigation on emergency innate lymphopoiesis that might be critical in controlling specific inflammatory insults.

## Materials and Methods

### Mice

C57BL/6 mice were bred locally. *Id2* ^Gfp/+^ (Rawlins *et al*.^42^) mice were purchased from Jackson Laboratories and bred locally. *Rag2* ^−/−^ *Il2Rγc* ^−/−^ mice were kindly provided by professor Klas Kärre (Karolinska Institutet, Stockholm). All mice were in a C57/BL/6 background and were used between 8 and 20 weeks of age. For tumor-mediated CHILPs expansion experiment, mice were injected with 2 × 10^6^ B16 or B16-Flt3L cells subcutaneously and two weeks after injection they were sacrificed for analysis. For Flt3L cytokine *in vivo* treatment, mice were intraperitoneally injected with 10 μg of recombinant Flt3L (eBioscience) for 10 consecutive days. Animals were maintained under specific pathogen-free conditions and handled according to protocols approved by the Stockholm Regional Ethics Committee and the responsible federal health Authorities of the State of Hamburg (Behörde für Gesundheit und Verbraucherschutz). All animal experiments were performed in accordance with national and institutional guidelines and regulations.

### Isolation of leukocytes

Colon and small intestine lamina propria cells were isolated as previously described^43^ with slight modifications. Briefly, intestines were harvested and placed in ice-cold PBS. After removal of residual mesenteric fat tissue, Peyer’s patches were excised (in the case of small intestine dissection) and the intestines were opened longitudinally. Tissues were then washed in ice-cold PBS and cut into 1-cm pieces. After 3 more washes in PBS, tissues were incubated on a shaking incubator at 37 °C for 30 minutes in 20 mL of HBSS containing 5% FCS, 5 mM EDTA, 1 mM DTT and HEPES 15 mM. Tissue pieces were washed with 20 mL PBS with 5%FCS and EDTA 1 mM followed by PBS with 1%FCS and 15 mM HEPES. Small intestine pieces were next digested in 10 ml of serum-free HBSS containing Liberase TL (0.15 mg/ml, Roche) and 0.1 mg/ml DNase I (Roche) at 37 °C at 600 rpm for 45 min. Cells were washed and passed through a 100-μm cell strainer. Cells were resuspended in 4.5 ml of 44% Percoll (Sigma Aldrich) and 2.3 mL of 67% Percoll were then underlaid. Percoll gradient separation was performed by centrifugation for 20 min at 600 × *g* at room temperature. Lymphoid fractions were collected at the interphase of the Percoll gradient, washed once, and resuspended in FACS buffer (5% FCS DPBS) or culture medium.

Lungs were minced with scissors and digested with 10 ml HBSS containing 0.15 mg/ml Liberase TL and 0.1 mg/ml DNase I at 37 °C at 600 rpm for 50 min. Percoll gradient centrifugation was then performed to enrich leukocytes.

Splenocytes single cell suspension was obtained by passing the tissue through a 70-µm cell strainer (Corning). After removal of muscle tissue, bones were crushed with a pestle and filtered through 70-μm cell strainer. The indicated absolute numbers of precursors in the bone marrow are calculated per one femur.

### Flow cytometry

Single cell suspensions were incubated for 15 min at 4 °C with Fc-blocking (CD16/32) antibody (eBioscience) prior to staining with fluorochrome-conjugated antibodies for 15 min at 4 °C. For staining of CHILPs in the BM, the following lineage cocktail was used: CD11c (N418), TER-119 (TER-119), CD3 (145-2c11), CD19 (6D5), CD11b (M1/70), NK1.1 (PK136) or in addition CD5 (53-7.3), B220 (RA3-6B2) and Gr-1 (RB6-8C5) were included. When using *Id2* ^Gfp/+^ mice, CHILPs were defined as Lin^−^ CD127^+^ Id2^+^ CD135^−^ α4β7^+^ CD25^−^. When using wild type C57BL/6 mice (as in DSS and Malaria experiments), CHILPs were defined as Lin^−^ CD127^+^ CD135^−^ α4β7^high^ CD25^−^. For intracellular transcription factors and cytokine staining, after cell surface staining, cells were incubated with Fixation and Permeabilization buffer (Foxp3 staining kit, eBioscience) for 30 min at 4 °C followed by staining for 20 min at room temperature with fluorochrome-conjugated antibodies against transcription factors. For IL-22 intracellular cytokine staining, small intestine cell suspensions were stimulated for 3 hours with 40 ng/ml of IL-23 (R&D Systems) in the presence of Golgi Stop (BD Biosciences). For IL-5 intracellular cytokine staining, small intestine cell suspensions were stimulated for 3 hours with PMA (100 ng/ml) and Ionomycin (1 μg/ml) in the presence of Golgi Stop (BD Biosciences).The following antibodies were purchased from eBioscience: α4β7 (DATK32), CD135 (A2F10.1), PLZF (Mags.21F7), IL-22 (IL22JOP), NKp46 (29A1.4), Eomes (Dan11mag) and GATA3 (TWAJ). The following antibodies were purchased from Biolegend: CD11c (N418), TER-119 (TER-119), CD3 (145-2c11), CD19 (6D5), CD127 (A7R34), T-bet (4B10), Gr-1 (RB6-8C5), B220 (RA3-6B2), CD5 (53-7.3), IL-5 (TRFK5), CD4 (RM4-5), CD45.2 (104) and CD45.1 (A20). The following antibodies were purchased from BD Biosciences: CD11b (M1/70), NK1.1 (PK136), CD25 (PC61), CD90 (53-2.1) APC, CCR6 (140706) and RORγt (Q31-378). DAPI or Live/Dead Fixable viability dyes (eBioscience) were used to exclude dead cells. All the experiments were acquired using FACS Canto II or FACS LSR Fortessa flow cytometers (BD Biosciences) and analyzed with FlowJo software (TreeStar).

### Cell sorting and *in vivo* differentiation

For *in vivo* differentiation experiments, CHILPs were highly purified by magnetic beads enrichment followed by FACS sorting. Briefly, single-cell suspension of leukocytes obtained from bones was stained with biotinylated antibodies against CD11b (M1/70, eBioscience), CD19 (MB19-1, Biolegend), TER-119 (TER-119, eBioscience). Stained cells were passed through LD depletion column (Miltenyi Biotec) according to manufacturer’s instructions. The enriched Lineage negative fraction was stained with fluorescently labeled antibodies (as described above) and then sorted using a BD FACSAria I or BD FACSAriaIII cell sorter (BD Biosciences). Highly purified CLP (Lin^neg^ CD127^+^ Id2^−^ CD135^+^) or CHILPs (Lin^neg^ CD127^+^ Id2^+^ CD135^−^ α4β7^+^ CD25^−^) from PBS or B16-Flt3L injected *Id2* ^Gfp/+^ donors were injected intravenously into *Rag2* ^−/−^ *Il2rg* ^−/−^ recipient mice. Each recipient mouse received between 150 to 2000 cells. Five to ten weeks after transfer, recipient mice were sacrificed and donor-derived cells were analyzed in the indicated organs. Engraftment index was calculated by dividing the number of ILCs retrieved in the host by the number of progenitors cells injected in the same mouse.

### Real-time PCR

For measurement of *Flt3l* transcripts in colonic tissue, 1 cm segment was excised and preserved in RNAlater at −80 °C. Tissues were homogenised by bead-beating (Precellys) followed by RNA isolation with RNAeasy Mini Kit (Qiagen) and reverse transcription with iScript RT Supermix (BioRad). Gene expression was analyzed by quantitative real-time PCR using iTaq Universal SYBR Green Supermix (BioRad) and fold changes were calculated relative to *Hprt* mRNA using 2-ddCt method^44^.

### Flt3L detection by ELISA

Flt3L in mouse serum was measured by ELISA with streptavidin-HRP reaction (substrate reagents and stop solution were purchased from R&D Systems). The following antibodies were used to perform ELISA: Flt3L capture antibody AF427 (R&D Systems) and Flt3L detection antibody BAF427 (R&D Systems). FLT3L in human plasma from healthy controls and IBD and malaria-infected patients was measured using an ELISA kit (R&D Systems DFK00).

### Malaria patient cohort

*Plasmodium falciparum* infected adults (*n* = 39) treated at Karolinska University Hospital, Stockholm, Sweden, were recruited at the time of diagnosis. The patients consisted of 16 Europeans and 23 immigrants from Sub-Saharan African countries (median age 35, range 22–62 years). All patients presented with fever and the majority (90%) had uncomplicated malaria, whereas 4 (10.2%) patients presented with severe malaria according to WHO criteria; 3 with circulatory shock and one with renal failure. Healthy Swedish adults (*n* = 28), with no prior history of malaria and no prior visits to malaria endemic areas, were included as controls (median age 32, range 20–59 years). On the day of diagnosis, venous blood was collected in EDTA and stored frozen as plasma and packed cells. Parasitemia was determined by conventional light microscopy as percentage of infected erythrocytes.

Written informed consent was provided by all study participants and the study was approved the Regional Ethical Review Board in Stockholm. All experiments were performed in accordance with the relevant guidelines and regulations.

### IBD patients’ cohort

Plasma from four different patient groups was collected and analyzed at the Gastromedical Center, Unit for Endoscopy at Karolinska University Hospital, Stockholm (Supplementary Table 1). Informed consent was provided by all study participants and the study was approved the Regional Ethical Review Board at Karolinska Institutet. All experiments were performed in accordance with the relevant guidelines and regulations. Subjects below 50 years with Lynch syndrome, an inherited disorder that increases the risk of colorectal cancer (*n* = 9), constituted the control group. Patients with active intestinal inflammation that were either newly diagnosed with IBD (*n* = 12) or diagnosed with IBD for longer than one year (*n* = 21) constituted the patient groups. The exact diagnosis of CD or UC, regular medication intake, age and gender were documented for every patient. Patients with tumors or other immune disease of the intestine and partial colectomy were excluded from the study. The inclusion criteria for IBD patients with active inflammation was if they did not take any substances besides 5-AZA within the last 3 months, for example no treatment with anti-TNF or thiopurines (including AZA treatment). Besides, patients also did not receive systemic corticosteroids within the last month.

Plasma was isolated after centrifugation at 1500 × g for 10 min of peripheral blood collected in heparin tubes. Plasma samples were aliquoted and stored frozen at −80 °C.

### Malaria mouse model

*Plasmodium berghei* ANKA infected red blood cells were stored in liquid nitrogen in a solution containing 0.9% NaCl, 4.6% sorbitol and 35% glycerol. Infection was performed as previously described^45^. Briefly, C57BL/6 mice were infected with *Plasmodium berghei* ANKA-infected red blood cells intraperitoneally (i.p.) and the blood was collected 5 to 6 days post-infection. 1 × 10^5^ infected red blood cells were then used to infect the experimental mice i.p. BM and serum of *Plasmodium berghei* ANKA -infected mice were collected at days 5, and 7 after infection. Later time points cannot be addressed since infected mice start to develop cerebral symptoms between day 8 and 9 after infection.

### Dextran sulfate sodium (DSS) administration

Mice were fed 2.5% (w/v) dextran sulfate sodium (DSS, MP Biomedicals; MW = 36,000–50,000) dissolved in drinking water *ad libitum* for 7 days, followed by 7 days of regular drinking water. Body weights were monitored every other day.

### Statistical analysis

Data were analyzed using GraphPad Prism Software 6.0d. p value was calculated using unpaired two-tailed Student’s *t*-test with 95% confidence interval when comparing two groups. Data were analyzed using one-way ANOVA with Bonferroni’s multiple comparisons test with 95% confidence interval when comparing more than two groups.

## Electronic supplementary material

Supplementary material
