# Supplementary material for: Flt3 ligand expands bona fide innate lymphoid cell precursors in vivo
Source: Sci Rep. 2018 Jan 9;8:154. doi: 10.1038/s41598-017-18283-0 (PMC5760642; doi:10.1038/s41598-017-18283-0)
Supplement: Supplementary file 1 — Supplementary material [file 41598_2017_18283_MOESM1_ESM.pdf]

Flt3 ligand expands bona fide innate lymphoid cell precursors in vivo

Sara M. Parigi, Paulo Czarnewski, Srustidhar Das, Christiane Steeg, Leonie Brockmann, Sara Fernandez-Gaitero, Victor Yman, Marianne Forkel, Charlotte Höög, Jenny Mjösberg, Lisa Westerberg, Anna Färnert, Samuel Huber, Thomas Jacobs & Eduardo J. Villablanca

Supplementary Material: Supplementary figure 1-3 and supplementary table 1.

Supplementary Figure 1

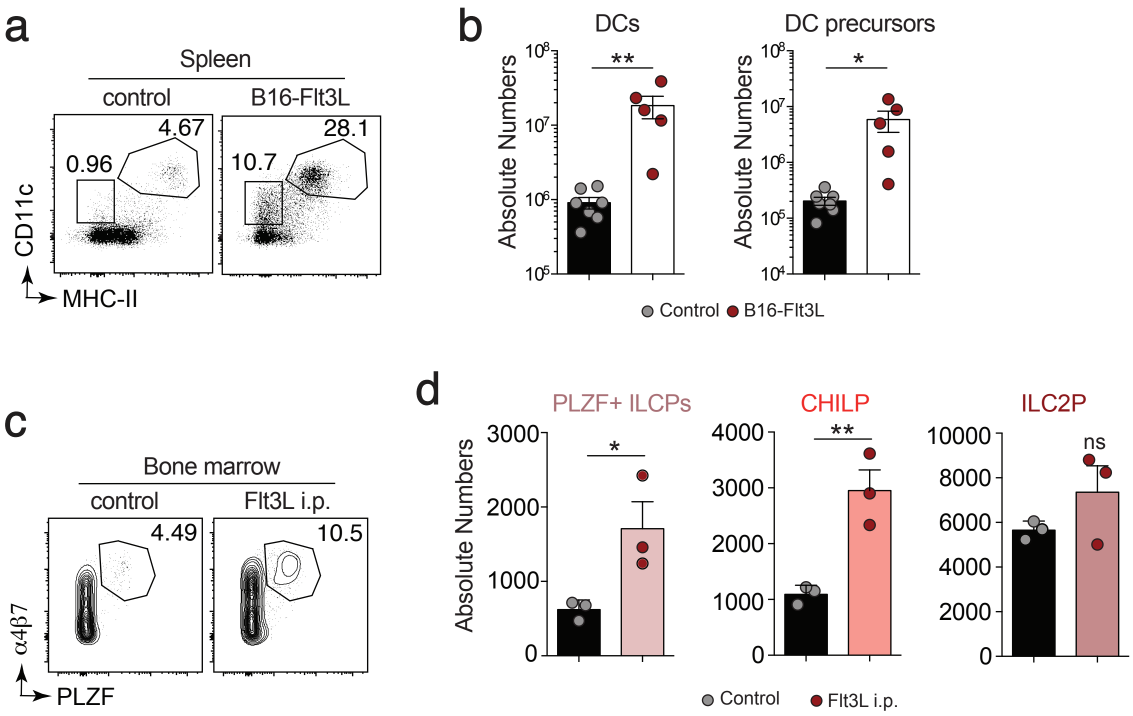

Supplementary Figure 1. (**a-b**) Mice were injected with 2x10^{6} B16-Flt3L or B16 as a control. Two weeks after tumor injection, differentiated DCs (CD11c^{+}MHC-II^{hi}) and DC precursors (CD11c^{+}MHC-II^{neg}) were analyzed in the spleen. Representative dot plots (**a**) and total number (**b**) of differentiated DCs (CD11c^{+}MHC-II^{hi}) and DC precursors (CD11c^{+}MHC-II^{neg}). Displayed representative dot plots show CD11c vs MHC-II after pre-gating in live cells CD45+ Lineage (CD19 and CD3) negative (*n*=5-7/group, 3 experiments). (**c-d**) Mice were injected with 10 μg of recombinant Flt3L intraperitoneally for 10 days or left untreated as controls. (**c**) Representative dot plots of bone marrow ILCP, defined as Lineage (CD3, CD19, CD5, B220, CD11c, CD11b, Gr-1, NK1.1, TER-119) negative CD135^{-} CD127^{+} PLZF^{+} α4β7^{+}. (**d**) Absolute numbers of bone marrow ILCP, CHILP and ILC2P. *p < 0.05; **p < 0.01; ns, not significant; Student's *t*-test. Error bars represent SEM in all panels.

Supplementary Figure 2

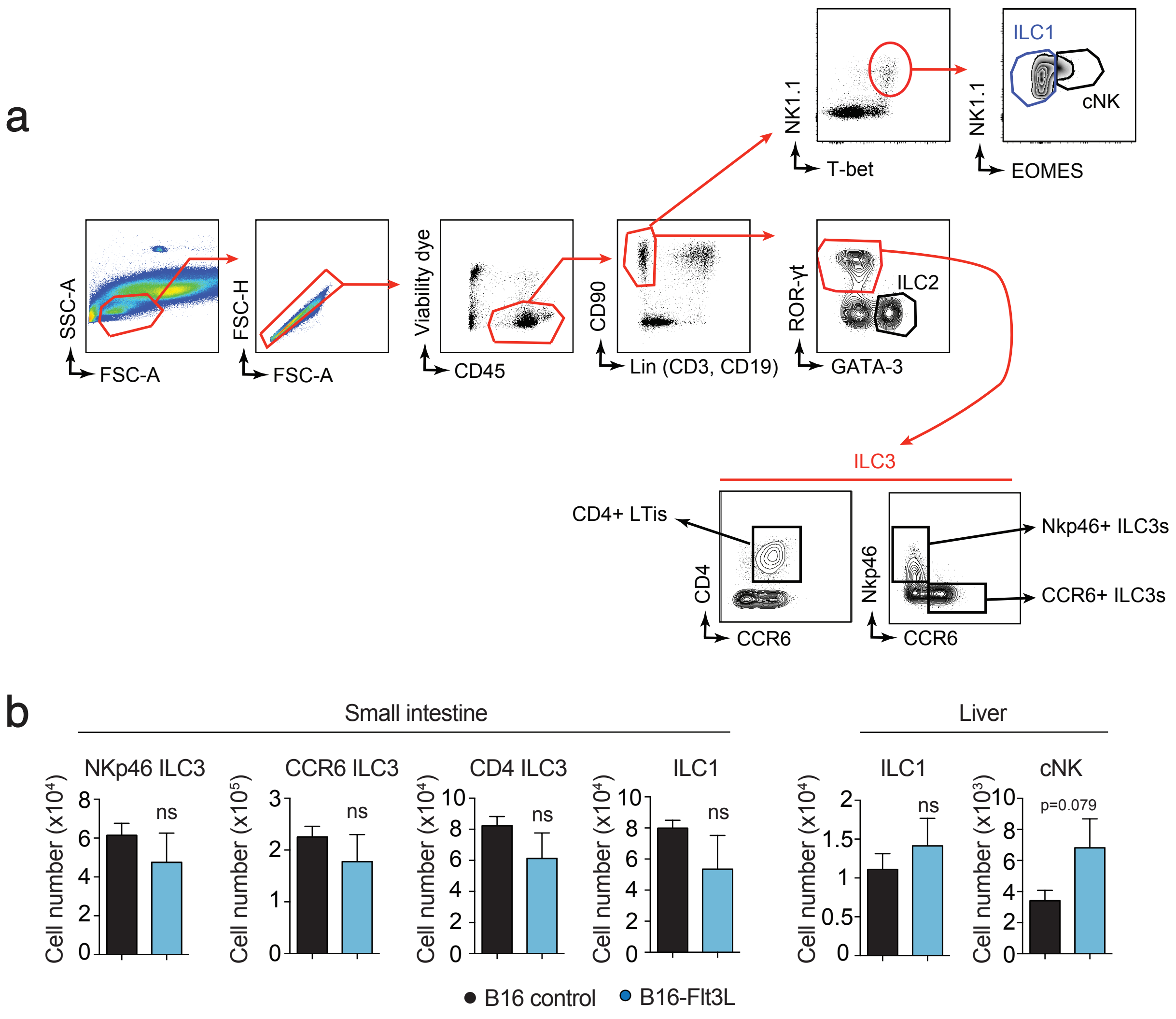

Supplementary Figure 2. (**a**) Gating strategy to analyze ILCs subsets from intestinal tissues. Same strategy was followed to analyze other tissues. Total ILCs were defined as viable $CD45^+CD90^+CD3^-CD19^-$. ILC1 were defined as NK1.1$^+$ T-bet$^+$ Eomes$^-$ ILCs; cNK as NK1.1$^+$ T-bet$^+$ Eomes$^+$; ILC2s as GATA-3$^+$ ILCs, and ILC3 as RORγt$^+$ ILCs. ILC3 subsets were analyzed based on the expression of NKp46, CCR6 and CD4. (**b**) Absolute numbers of the indicated ILC subsets in the small intestine lamina propria and liver of mice injected B16-Flt3L or B16 cells as controls. ns, not significant; Student's t-test. Error bars represent SEM in all panels.

Supplementary Figure 3

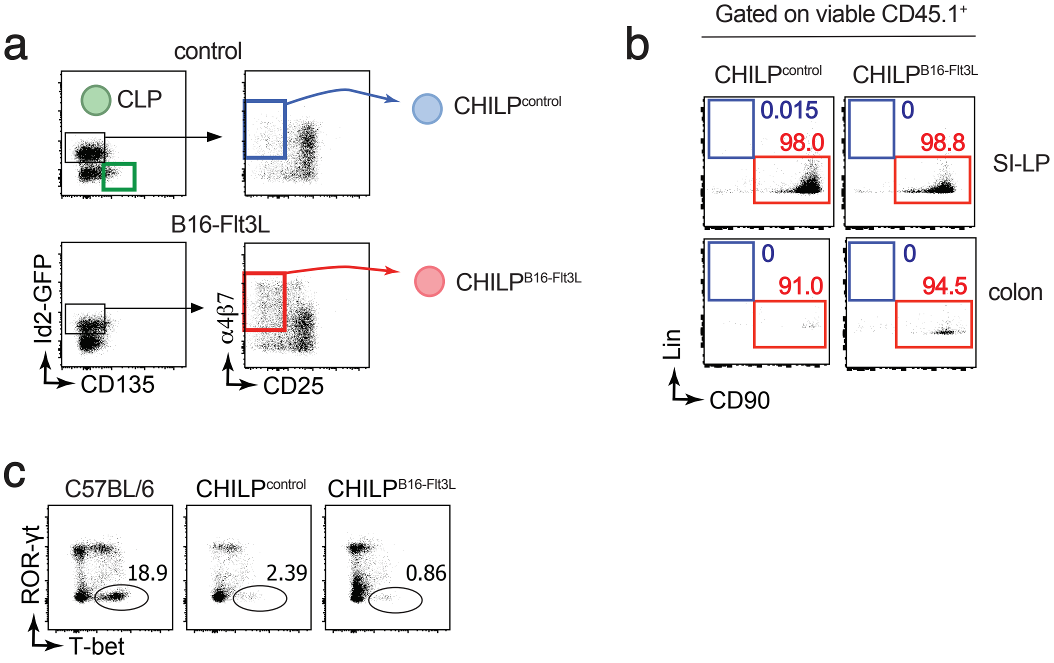

Supplementary Figure 3. (**a**) Definition of CLP and CHILP to be adoptively transferred into lymphopenic recipient mice. Representative dot plots showing the FACS-sorted populations before sorting. (**b**) Representative dot plots of donor-derived cells in the small and large intestine lamina propria of CD45.2$^+$ lymphopenic mice adoptively transferred with CD45.1$^+$ CHILP^{control} or CHILP$^{B16\text{-}Flt3L}$ 8 weeks after transfer. Displayed plots are gated on viable CD45.1$^+$ cells. Gated in red are CD90$^+$ Lineage (Lin: CD11c, Gr-1, CD64, TER-119, CD19) negative cells; gated in blue are CD90$^-$ Lin$^+$ cells. (c) Representative dot plots of donor-derived T-bet$^+$RORγt^{neg} ILCs in the small intestine lamina propria of lymphopenic mice adoptively transferred with CHILP^{control} or CHILP$^{B16\text{-}Flt3L}$. Displayed plots are gated on viable CD45.1$^+$CD90$^+$CD3$^-$ cells. Small intestine ILCs from a wild-type C57BL/6 mouse are shown as a control.

Supplemental Table 1.

Diagnosis	Comments	G	Age(years)	Medication
Healthy control	non-IBD no sypmtoms	M	33	-
Healthy control	non-IBD no sypmtoms	M	53	-
Healthy control	non-IBD no sypmtoms	F	46	-
Healthy control	non-IBD no sypmtoms	F	41	-
Healthy control	non-IBD no sypmtoms	M	29	-
Healthy control	non-IBD no sypmtoms	M	43	-
Healthy control	non-IBD no sypmtoms	F	32	-
Healthy control	non-IBD no sypmtoms	F	35	-
Healthy control	non-IBD no sypmtoms	F	44	-
new IBD	new CD	F	21	-
new IBD	new CD	F	49	-
new IBD	new CD	F	38	-
new IBD	new CD	M	32	-
new IBD	Suspect new onset UC in relapse	M	30	-
new IBD	Suspect new onset UC in relapse	M	23	-
new IBD	new UC	M	56	asacol
new IBD	new UC	F	20	mesasal
new IBD	new UC	M	34	-
new IBD	new UC	F	21	-
new IBD	UC >1year or Crohn`s >1year	F	62	-
new IBD	new UC	F	38	-
Chronic CD	Crohn`s >1 year	F	30	colazid
Chronic CD	Crohn`s >1 year	M	36	asacol
Chronic CD	Crohn`s >1 year	F	53	azathioprim, salazopyrin
Chronic CD	new CD	M	78	salazopyrin
Chronic CD	Crohn`s >1 year	F	34	-
Chronic CD	Crohn`s >1 year	F	44	-
Chronic CD	Crohn`s >1 year	M	20	salazopyrin
Chronic CD	Crohn`s >1 year	M	48	asacol
Chronic CD	CD>1year	F	30	-
Chronic CD	CD>1year	M	52	asacol
Chronic UC	UC >1year	M	62	-
Chronic UC	UC >1year	M	39	pentasa, asacol
Chronic UC	UC >1year	M	23	asacol
Chronic UC	UC >1year	M	22	-
Chronic UC	UC >1year	F	57	-
Chronic UC	UC >1year	F	39	-
Chronic UC	UC >1year	M	40	asacol
Chronic UC	Crohn`s >1 year or UC >1year	M	34	colazid
Chronic UC	UC >1year	F	46	-
Chronic UC	UC >1year	M	59	salazopyrin
Chronic UC	UC >1year	F	40	asacol

Supplemental Table 1. Information of patients recruited for plasma cytokine measurements. UC indicates ulcerative colitis, CD indicates Crohn's disease, IBD indicates inflammatory bowel diseases, G indicates Gender, F indicates female, M indicates male.
